# Inhibition of HMGB1 Ameliorates the Maternal-Fetal Interface Destruction in Unexplained Recurrent Spontaneous Abortion by Suppressing Pyroptosis Activation

**DOI:** 10.3389/fimmu.2021.782792

**Published:** 2021-12-23

**Authors:** Damin Zhu, Huijuan Zou, Jinxian Liu, Jing Wang, Cong Ma, Jiaqian Yin, Xiaoqing Peng, Danyang Li, Yulu Yang, Yu Ren, Zhiguo Zhang, Ping Zhou, Xiangyan Wang, Yunxia Cao, Xiaofeng Xu

**Affiliations:** ^1^ Reproductive Medicine Center, Department of Obstetrics and Gynecology, the First Affiliated Hospital of Anhui Medical University, Hefei, China; ^2^ Department of Obstetrics and Gynecology, Chaohu Hospital of Anhui Medical University, Chaohu, China; ^3^ National Health Commission Key Laboratory of Study on Abnormal Gametes and Reproductive Tract (Anhui Medical University), Hefei, China; ^4^ Key Laboratory of Population Health Across Life Cycle (Anhui Medical University), Ministry of Education of the People's Republic of China, Hefei, China; ^5^ Anhui Province Key Laboratory of Reproductive Health and Genetics (Anhui Medical University), Hefei, China; ^6^ Biopreservation and Artificial Organs, Anhui Provincial Engineering Research Center, Anhui Medical University, Hefei, China; ^7^ Anhui Provincial Institute of Translational Medicine(Anhui Medical University), Hefei, China

**Keywords:** maternal-fetal Interface, high mobility group B-1, macrophage, pyroptosis, URSA

## Abstract

Recurrent spontaneous abortion (RSA) is a common complication of pregnancy that affects the physical and mental health of pregnant women, and approximately 50% of the mechanisms are unclear. Our previous studies have found that high mobility group box 1 (HMGB1) molecules are highly expressed at the maternal-fetal interface of unexplained recurrent spontaneous abortion (URSA) patients. The purpose of this study was to further detect the expression of HMGB1 and pyroptosis in decidual tissue of URSA patients, and explore the potential mechanism of the protective role of HMGB1 in URSA patients and mouse model. The decidua tissues of 75 URSA patients and 75 women who actively terminated pregnancy were collected, and URSA mouse models were established and treated with HMGB1 inhibitor-aspirin. The expression of HMGB1, and their receptors (RAGE, TLR2, TLR4), pyroptosis-associated proteins (NLRP-3, caspase-1, GSDMD) and NF-κB was examined at the maternal-fetal interface of human and mouse. Our study found that HMGB1, NLRP-3, Caspase-1, GSDMD, RAGE, TLR2 and TLR4 were highly expressed and NF-κB signaling pathway were activated in the decidua tissue of URSA group. Moreover, immune cell disorder and co-localization of HMGB1 and macrophages were found at the maternal-fetal interface of URSA mice. However, HMGB1, TLR2, TLR4, NF-κB, and pyroptosis-associated proteins can be down-regulated by administering low-dose aspirin. These data may indicate that highly expressed HMGB1 was actively secreted by macrophages and then activated pyroptosis through the TLR2/TLR4-NF-κB pathway to cause aseptic inflammation, leading to the occurrence and development of URSA. Moreover, low-dose aspirin can reduce HMGB1 protein levels of serum and decidual in URSA.

## Introduction

Two or more consecutive or discontinuous losses of pregnancy products before 24 weeks with the same sex partner are referred to as recurrent spontaneous abortion (RSA) or recurrent pregnancy loss (RPL) (1, 2). In recent years, the incidence of RSA has been relatively high, affecting approximately 1% of women of childbearing age (3). The risk of RSA also increases further with the number of miscarriages, reaching about 40% after three consecutive miscarriages, and the prognosis is poor with increasing age (4). This not only affects the physical and mental health of pregnant women, but also brings heavy burdens and mental pressure to their families. The etiology of RSA include genetic abnormalities, structural abnormalities, infection, endocrine abnormalities, immune dysfunction, antiphospholipid syndrome, and thrombophilic disorders, etc (5). However, the causes in nearly 50% of RSA patients are still unclear, and the clinical diagnosis is called unexplained recurrent spontaneous abortion(URSA) (6). Fetuses as allografts are able to grow and develop normally without rejection within the mother, which depends on the process of a constant reciprocal balance of maternal immune rejection or immune tolerance to the fetus (7). Abnormal immune tolerance at the maternal-fetal interface could be one of the important pathogenesis of URSA (8).

High mobility group box-1 (HMGB1) is a highly conserved non-histone DNA-binding protein widely distributed in the nucleus of eukaryotic cells, characterized by the low molecular weight and rapid mobility in the gel electrophoresis (9, 10). HMGB1, a typical damage-associated molecular pattern (DAMP) molecule, can be actively or passively released into the extracellular space (10), which binds to its receptor ligands RAGE, TLR, CXCR4, TIM-3, CD24, NMDAR, and haptoglobin to exert the proinflammatory role (11, 12). Inflammation plays an important role in embryo implantation, development, and endometrial decidualization (13). Low level of HMGB1 can cause the physiological inflammatory response, recruit immune cells, and promote the proliferation and differentiation of decidual stromal cells that are required for nomal pregnency (13). However, high levels of HMGB1 in the uterine cavity were associated with pregnancy failure (13). Recent studies have found that HMGB1 was highly expressed in villous and decidual tissues of RSA patients (14, 15), moreover, animal experiments have also shown that excessive administration of HMGB1 leaded to the failure of pregnancy (16).

Pyroptosis, which has been extensively studied recently, is a lytic and inflammatory programmed cell death pathway different from apoptosis (17). It is involved in the occurrence and development of a variety of diseases in multiple organs of the body. Pyroptosis directly assembles the inflammasome (NLRP3) by damage-associated molecular pattern (DAMP) and activates the cysteine protease caspase-1, allowing the translocation of the N-terminal domain fragment of the GasderminD (GSDMD) protein to the cell membrane to form β-barrel transmembrane pores (17). Hence, the plasma membranes were rapidly ruptured to release cellular contents and a large number of inflammatory factors, and send proinflammatory signals to adjacent cells to recruit inflammatory cells and induce an inflammatory response, eventually causing cell death (18).

HMGB1 induced hepatocyte pyroptosis has been shown to lead to aseptic inflammation and liver injury (19). But the pyroptosis mediated by HMGB1 has never been investigated in URSA patients. Moreover, our previous studies have found that the decidua tissue of URSA patients highly expresses HMGB1 (20), and the peripheral blood HMGB1 expression of URSA patients taken aspirin of the most effective inhibitors of HMGB1 is decreased (15, 21). However, the specific mechanism is not clear. Therefore, this study aimed to investigate the release mechanism of HMGB1 in URSA and explore the effect of blocking HMGB1 on URSA. This study would provide a theoretical basis for clinical treatment of URSA patients.

## Materials and Methods

### Human Sample

From September 2017 to December 2020, 140 pregnant women who were younger than 35 years old with a history of two or more unexplained miscarriage were recruited from the Department of Obstetrics and Gynecology of the First Affiliated Hospital of Anhui Medical University in this study. Exclusion criteria included anatomical abnormalities of the genital tract, endocrine abnormalities, immune abnormalities, coagulation abnormalities, karyotype abnormalities in couples, autoantibody abnormalities such as anticoagulant lipid antibodies, thyroid dysfunction, genetics, and infection. Pregnant women underwent a vaginal ultrasound examination every two weeks. When the fetal heart rate was found to disappear, it was defined to terminate the pregnancy and a villus chromosome test was performed. Decidua tissues during artificial abortion were then collected, and only 75 patients were enrolled for follow-up study after excluding the decidua tissues with abnormal chorionic chromosome detection. In the control group, 75 pregnant women from the same hospital voluntarily were chosen to terminate the pregnancy by abortion, from which the decidual tissue were collected. The biological sample study was approved by the Medical Ethics Committee of the First Affiliated Hospital of Anhui Medical University (Ethics approval number: PJ2018-02-09; Clinical Trial Registration number: ChiCTR1800015403).

### Animals

Female CBA/J mice and male DBA/2J mice (8-10 weeks old) were purchased from Beijing Huafukang Biotechnology. Male BALB/c mice (8-10 weeks old) were purchased from the Experimental Animal Center of Anhui Medical University. All mice were adaptively fed for one week at a standard environment of constant temperature of 21-23°C and humidity of 50-60%, with autoclaved sterile water and diet. All animal experiments were controlled specific pathogen-free conditions at the Experimental Animal Center of Anhui Medical University. All experimental protocols were approved by the Experimental Animal Ethics Committee of Anhui Medical University (ethics number: LLSC20201138).

### Establishment and Intervention of URSA Mouse Model

All female mice were randomly divided into three groups. Female CBA/J mice mated with male BALB/c mice as the control group. Female CBA/J mice mated with male DBA/2J mice as the URSA group. And aspirin-treated female CBA/J mice mated with male DBA/2J mice as the aspirin treatment group (URSA+ASPL). Female mice in the aspirin treatment group were given aspirin-containing drinking water (30 ug/ml) for 7 days before mating and 7 days after vaginal plug were observed, which all drinking water was changed every other day (22, 23). The control and URSA groups were added with the same content of normal saline. For successful mating, two females and one male were stayed together all night, and females with vaginal plug detected the next morning were recorded as gestational day (GD) 0. Subsequently, the food intake, activity, vaginal bleeding, and bodyweight of mice in each group were observed daily. The mice were euthanized on GD14. The uterus, embryos, placenta, and decidua were separated, and all the tissues were fixed with 4% paraformaldehyde and stored at -80°C. At the same time, the number of total fetuses and viable fetuses were counted. The fetuses were small and dark, accompanied by necrosis and hemorrhage as absorbed fetuses. The absorption rate of fetuses was calculated as the number of absorbed fetuses/number of total fetuses × 100% (24, 25).

### H&E Staining

The decidual tissues from mice were fixed with 4% paraformaldehyde for 24-48 hours. Then all samples were dehydrated with gradient alcohol, embedded in paraffin, and cut into 3-μm thick sections. Paraffin sections were deparaffinized with xylene and alcohol, stained with hematoxylin for 3 minutes and eosin for 2 minutes, and then mounted with resin. Finally, all sections were observed under the microscope.

### Immunohistochemistry Staining

The paraffin sections of decidua harvested at gestational day (GD) 14. They were baked in an oven at 60°C for 2 hours and then dewaxed with xylene and gradient alcohol. Then the slices were penetrated with 2% Triton solution for 20 minutes before being repaired with sodium citrate antigen retrieval solution. After blocking with normal goat serum working fluid for 30 min, the slices were incubated with HMGB1(1:400 dilution; Abcam, Cambridge, UK), and NF-κB (1:500 dilution; Cell Signaling TECHOLOGY, MA, USA) at 4°C overnight. Then all samples were incubated with the Streptavidin/Peroxidase HistostainTM -Plus Kits (ZSGB-BIO, Beijing, China), and stained with DAB. The positive cells were observed under a Zeiss (Carl Zeiss AG, Oberkochen, Germany) microscope after the sections were sealed with resin.

### Western Blotting Assay

Mouse decidua and human decidual tissue were extracted with lysis buffer, and the protein concentration was detected with a BCA protein quantification kit (Beyotime, Shanghai, China). The total lysate was separated by 10%-12% sodium dodecyl sulfate-polyacrylamide gel electrophoresis (SDS-PAGE) and transferred to a polyvinylidene fluoride (PVDF) membrane. The membranes were incubated overnight with HMGB1 (1:10000 dilution; Abcam, Cambridge, UK), GSDMD (1:500 dilution; Affinity, USA), caspase-1 (1:1000 dilution; Abcam, Cambridge, UK), NLRP3 (1:200 dilution; NOVUS, USA), TLR2 (1:500 dilution; Abcam, Cambridge, UK), TLR4 (1:500 dilution; Abcam, Cambridge, UK), RAGE (1:1000 dilution; Abcam, Cambridge, UK) antibodies in a shaker at 4°C overnight, β-actin (1:1000 dilution; Abcam, Cambridge, UK) was used as an internal reference control. Membranes were incubated with secondary antibodies for 2 hours after washing the next day, and the ECL detection kit (Pierce Biotechnology, Rockford, USA) was then used for signal detection.

### Immunofluorescence Staining

Subcellular localization of HMGB1 and F4/80 or CD68 proteins in decidua was identified by using immunofluorescent staining. The steps before blocking were the same as immunohistochemistry. After blocking with 10% donkey serum for 2 hours, they were incubated with HMGB1(1:400 dilution; Abcam, Cambridge, UK), and F4/80 (1:100 dilution; Invitrogen, MA, USA) or CD68(1:100 dilution; GeneTex, CA, USA) at 4°C overnight. The next day, sections were allowed to return to room temperature and incubated with anti-rabbit IgG-594 (1:1000 dilution; Jackson, PA, USA) and anti-rat FITC (1:50 dilution; ZSGB-BIO, Beijing, China) for 2 hours. Then stain the cell nucleus with Hoechst 33342 (1:500 dilution; Thermo, USA.) for 5 minutes, and add a fluorescence quencher to observe under a fluorescence microscope (Carl Zeiss AG, Oberkochen, Germany).

### ELISA

Mouse blood was collected by eyeball removal and allowed to stand at room temperature for 2 hours, centrifuged at 3000 rpm for 10 minutes, and then the supernatant was aspirated and stored at −80°C until use. Then the HMGB1 concentration in the mice serum was further detected by the ELISA (CLOUD-CLONECORP, Wuhan, China), assay according to the manufacturers’ instructions.

### Statistical Analysis

Statistical analysis was performed using SPSS 22.0 and ImageJ, and the results were expressed as mean ± SEM. Differences between multiple groups were analyzed using analysis of one-way analysis of variance followed by Bonferroni correction, and the difference between the two groups were compared with the paired *t*-test. Values of *p* < 0.05 was considered statistically significant.

## Results

### Up-regulation of HMGB1 and Pyroptosis-Associated Proteins in Decidual Tissue of URSA Patients

**Figure 1:**
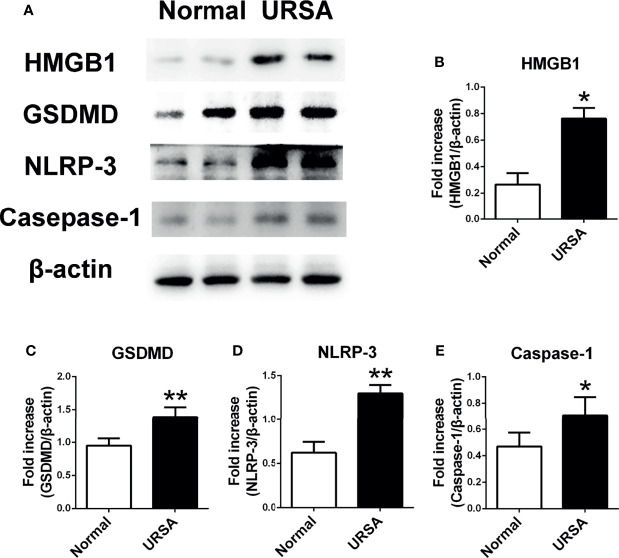
The expression of HMGB1 and pyroptosis-associated proteins GSDMD, NLRP-3, and Caspase-1 in decidual tissue of URSA patients. **(A)** HMGB1, GSDMD, NLRP-3, and Caspase-1 protein were detected using Western blotting. **(B)** Quantitative data analysis for HMGB1. **(C)** Quantitative data analysis for GSDMD. **(D)** Quantitative data analysis for NLRP-3. **(E)** Quantitative analysis for Caspase-1. All the results were representatives of three independent experiments and data were expressed as mean ± SEM. (n=20/group). *P < 0.05, **P < 0.01 compared to the control.

Decidual tissue was obtained from patients to investigate the expression of HMGB1 protein and pyroptosis-associated proteins. Consistent with our previous report, the protein expression of HMGB1 in the decidual tissue from the URSA patients (URSA group) was higher than that of the control (normal group) (**Figures 1A, B**). GSDMD, NLRP-3, and caspase-1 proteins were also up-regulated in the URSA group (**Figures 1A, C–E**). The above results indicated that HMGB1 and pyroptosis might be associated with URSA.

### Inhibition of HMGB1 Attenuated Inflammatory Responses at the Maternal-Fetal Interface in URSA Mice

**Figure 2:**
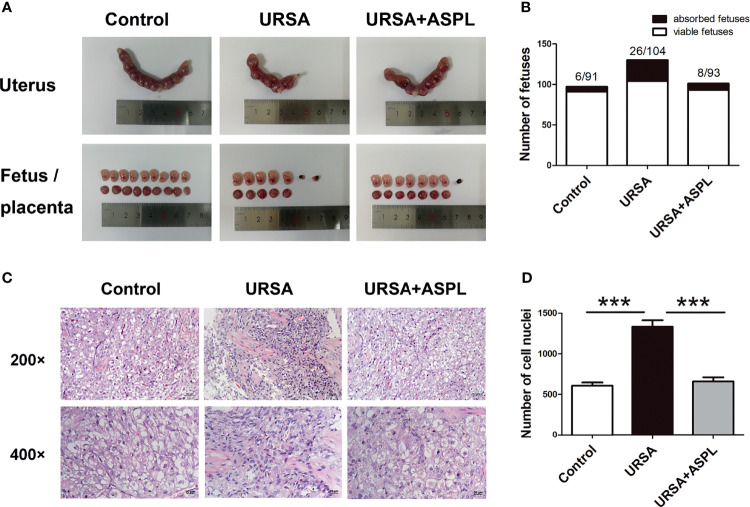
Uterine anatomy and pathological changes of decidua in mice. **(A)** Left and right uterine horns, fetus, and placenta from normal pregnant mice, aborted mice, and drug-treated mice. **(B)** The number of fetuses. **(C)** Decidua of the control group, URSA group, and URSA+ASPL group were stained by H&E staining. **(D)** Quantitative assessment of infiltrating cells in decidua by counting the number of cell nuclei in H&E staining. The results were representatives of three independent experiments and data were expressed as mean ± SEM. ***P < 0.001. Scale bar represents 50 μm and 20 μm. (n = 6/group).

To explore the pathogenesis of URSA, we established a mouse model of URSA. The uterus, fetus, and placenta of the three groups of mice were shown in **Figure 2A**. Compared to control group, some fetuses and placenta in URSA group were found to be small in size and darker in color. Aspirin is utilized as an inhibitor drug against HMGB1. Interestingly, the situation of miscarriage in the aspirin treatment group was better than that in the URSA group. The number of fetuses and embryo resorption rate were shown in **Figure 2B** and **Table 1**. The embryo resorption rate was 6.19% in the control group, 20.00% in the URSA group, and 7.92% in the aspirin group. The URSA group had a higher embryo absorption rate than the control group (*p*<0.01), indicating that the abortion model was successfully constructed. Treatment with aspirin reduced the rate of miscarriage compared to the URSA group (*p*<0.05). These results indicated that mouse model of URSA was established successful, and aspirin could reduce embryonic absorption rate in URSA group. Meanwhile, H&E staining showed that the infiltrating cells in the decidual tissue of the URSA group were significantly more than those of the control group, the difference was statistically significant (*p*<0.001), accompanied by disorganized cells and fragmented nuclei (**Figures 2C, D**). However, aspirin treatment could ameliorate the inflammatory response at the maternal-fetal interface in URSA mice, and the difference was statistically significant (*p*<0.001).

**Table 1:** The absorption rate among the three groups.

	Control	URSA	URSA+ASPL
Number of animals	11	14	12
Number of viable fetuses	91	104	93
Number of absorbed fetuses	6	26	8
Number of total fetuses	97	130	101
Absorption rate of fetuses (%)	6.19% (6/97)	20.00% (26/130)^a^	7.92% (8/101)^b^

Data were analyzed using the Chi-square. ^a^P < 0.01 compared with the Control group, ^b^P < 0.05 compared with the URSA group.

### Treatment With Aspirin Down-Regulated the Level of HMGB1

**Figure 3:**
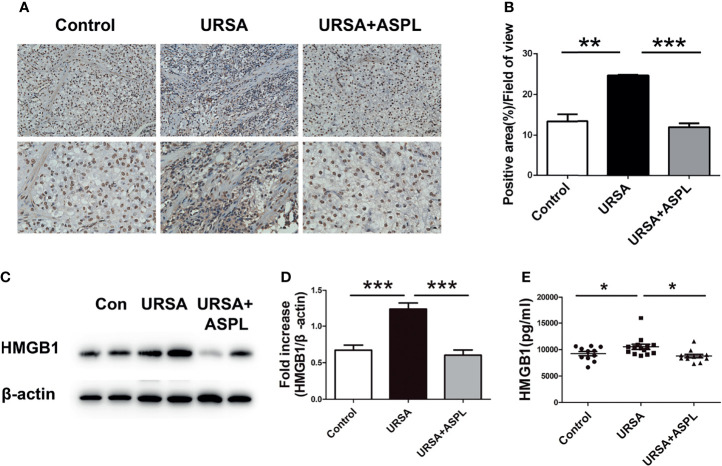
The expression of HMGB1 in mouse decidual tissue. **(A)** The expression of HMGB1 in mouse decidual tissue was investigated by immunohistochemistry staining. Scale bar represents 50 μm and 20 μm. (n = 6/group). **(B)** Quantitative assessment of the HMGB1 positive staining area in decidua using ImageJ software. **(C)** The protein levels of HMGB1 were identified by Western blot analysis. **(D)** The quantification data analysis of HMGB1 in the mouse decidual tissues. Data were expressed as mean ± SEM. (n = 6/group). **(E)** The serum HMGB1 levels of mice among control group, URSA group, and URSA+ASPL group were analyzed by ELISA. All the results were representatives of three independent experiments and data were expressed as mean ± SEM. *P < 0.05, **P < 0.01, ***P < 0.001 (Chart D n = 11 in control group; n = 14 in URSA group; n = 11 in URSA+ASPL group).

Our previous studies have proved that HMGB1 plays an important role in URSA patients (15). To verify the role of HMGB1 in the URSA mouse model, we examined the expression of HMGB1 in the decidual tissues of URSA mice by immunohistochemical staining. As shown in **Figures 3A, B**, compared with the control group, not all positive staining in the URSA group was in the nucleus, but there was a large amount of positive staining in the cytoplasm and extracellular. In the aspirin treatment group, HMGB1 was still expressed in the nucleus. The above difference was statistically significant (*p*<0.01). Biological effects only occur when HMGB1 is expressed outside the nucleus. Western blot detection showed that the expression level of HMGB1 protein was higher in the URSA group than in the control group, and decreased in the aspirin group (**Figures 3C, D**). These results indicated that HMGB1 was involved in the inflammatory response of URSA decidual tissue, which caused immune disorders. However, aspirin ameliorated HMGB1-induced immune disturbance at the maternal-fetal interface in URSA mice. Finally, the levels of HMGB1 in sera of mice in the control group, URSA group, and aspirin group were detected by ELISA (**Figure 3E**). The results showed that the serum HMGB1 expression level in the URSA group was higher than that in the control group, which was significantly lower after aspirin treatment. It was indicated that aspirin treatment could inhibit the expression of HMGB1 in URSA mice.

### HMGB1 Derived From Decidual Macrophages at the Maternal-Fetal Interface of URSA

**Figure 4:**
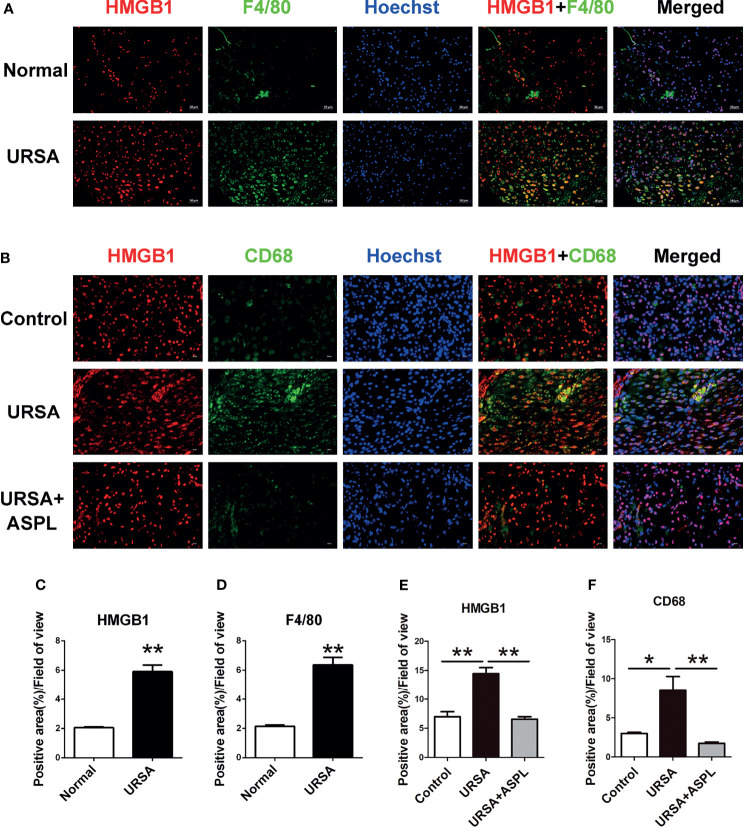
The colocalization between macrophages (F4/80^+^ or CD68^+^) and HMGB1 at the maternal-fetal interface. Double immunofluorescent staining of F4/80 and HMGB1 in human decidua **(A)** and CD68 and HMGB1 in mouse decidua **(B)**. Quantitative assessment of the HMGB1 **(C)** and F4/80 **(D)** positive staining area in human decidua using ImageJ software. Quantitative assessment of the HMGB1 **(E)** and CD68 **(F)** positive staining area in mouse decidua using ImageJ software. HMGB1 was labeled with red fluorescent reagent. F4/80 and CD68 were labeled with green fluorescent reagent. The nucleus was stained blue with Hoechst. Scale bar represents 50 μm (human, n = 20/group) and 20 μm (mouse, n = 6/group). All the results were representatives of three independent experiments, and data were expressed as mean ± SEM. *P < 0.05, **P < 0.01.

Double immunofluorescence staining was used to verify whether HMGB1 is released by macrophages at the maternal-fetal interface. F4/80 and CD68 are well-known surface-specific markers of macrophages. As shown in **Figure 4A**, the expression of HMGB1 (red) and F4/80 (green) was higher in the URSA group than that in the control group, and the difference was statistically significant (**Figures 4C, D**), and there were more HMGB1 and F4/80 double positive cells emerged. It was suggested that macrophage-derived HMGB1 was abundantly expressed in the nucleus as well as in the cytoplasm and extracellular space in the decidual tissue of URSA patients. This was also verified in mouse decidual tissue. As shown in **Figure 4B**, HMGB1 (red) and CD68 (green) double-positive cells were abundantly expressed in the URSA group, whereas this double-positive cell expression was reduced in the aspirin group, and the difference was statistically significant (**Figures 4E, F**). These indicated that macrophages in the decidual tissue of URSA could secrete HMGB1 actively, whereas aspirin could down-regulate the expression of macrophage-derived HMGB1.

### The Altered HMGB1 Related Receptors and NF-κB Signaling in Abortion Tissue Suffered From URSA

**Figure 5:**
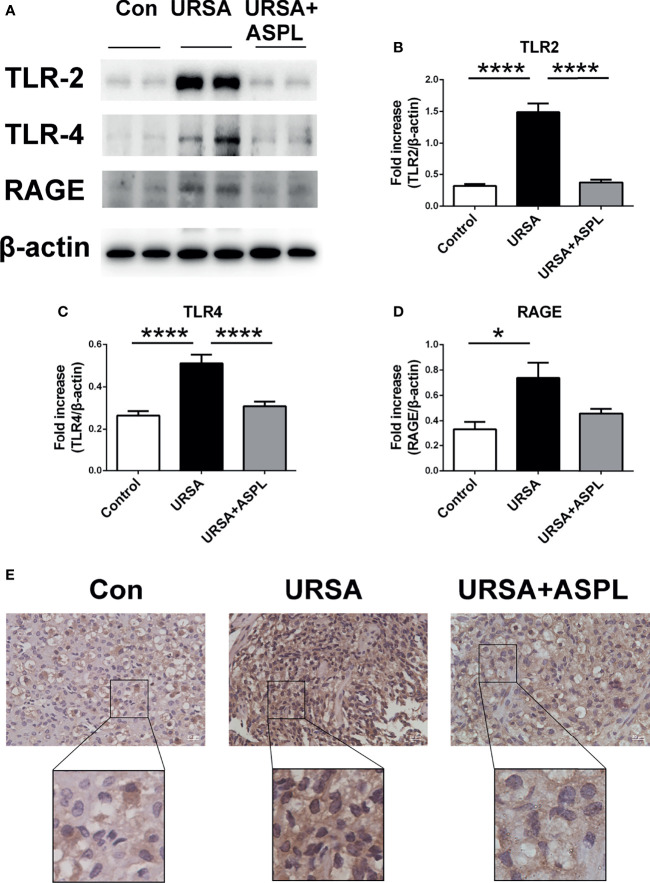
The analysis of TLR2, TLR4, RAGE, and NF-kB p65 in mouse decidua. **(A)** The protein levels of TLR2, TLR4, and RAGE were identified by Western blot analysis. **(B)** Quantitative data analysis for TLR2. **(C)** Quantitative data analysis for TLR4. **(D)** Quantitative data analysis for RAGE. (n = 6/group). **(E)** The expression of NF-κB p65 was detected by immunohistochemistry in decidua among control group, URSA group, and URSA+ASPL group. Scale bar represents 20 μm. (n = 6/group). All the results were representatives of three independent experiments and data were expressed as mean ± SEM. *P < 0.05, ****P < 0.0001.

To investigate whether HMGB1 expressed in decidual tissue was involved in receptors (RAGE/TLRs) and NF-κB intracellular signaling pathways, we measured the protein levels of RAGE, TLR2, and TLR4 in mice of three groups by western blotting. The protein expression of HMGB1 receptors RAGE, TLR2, and TLR4 in the URSA group were significantly up-regulated compared with the control group, while the expression were significantly down-regulated in the aspirin group (**Figures 5A–D**). Meanwhile, the location of NF-κB in the three groups was detected by immunohistochemical staining. Low levels of NF-κB p65 were concentrated in the cytoplasm and nucleus in the control group. While in the URSA group, there was an intense immune response to NF-κB p65 in both the nucleus and cytoplasm of mice decidua. The aspirin group was similar to the control group (**Figure 5E**). Therefore, the results showed that the HMGB1 related receptors (RAGE/TLR2/TLR4) were up-regulated and NF-κB signaling were activated in the decidua tissue of URSA group, and their expression could be down-regulated after aspirin intervention. Thus, we suggested that the HMGB1-TLRs/RAGE-NF-KB pathway may be played an important role in the maternal-fetal interface of URSA, and the inhibition of HMGB1 could block this pathway.

### Inhibition of HMGB1 Down-Regulated Pyroptosis-Associated Proteins at the Maternal-Fetal Interface in URSA

**Figure 6:**
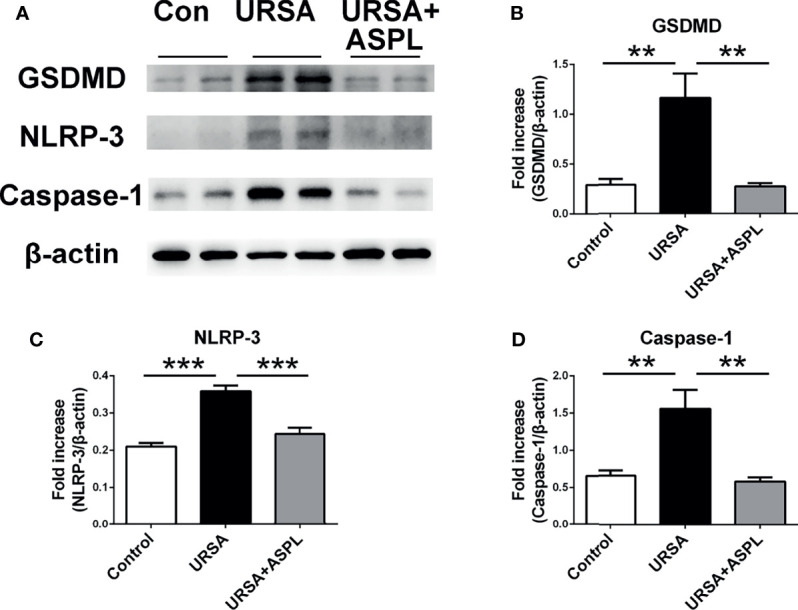
The analysis of GSDMD, NLRP-3, and Caspase-1 in mouse decidua. **(A)** The protein levels of GSDMD, NLRP-3, and Caspase-1 were identified by Western blot analysis. **(B)** Quantitative data analysis for GSDMD. **(C)** Quantitative data analysis for NLRP-3. **(D)** Quantitative data analysis for Caspase-1. All the results were representatives of three independent experiments and data were expressed as mean ± SEM. **P < 0.01, ***P < 0.01. (n = 6/group).

To determine the expression of pyroptosis-associated proteins in mouse decidual tissues, we examined the expression of GSDMD, NLRP-3, and caspase-1 with western blot assay. As shown in **Figure 6**, the expressions of GSDMD, NLRP-3, and caspase-1 were consistent with those in human decidua tissue, and the URSA group had higher expression than that of the control group (**Figures 6A–D**). However, the expression level of these proteins were down-regulated in the aspirin group. It was suggested that aspirin could intervene in the NLRP3-caspase1-GSDMD signaling pathway, and indicating that aspirin could suppress the activation of pyroptosis.

## Discussion

The present study found that HMGB1, GSDMD, NLRP3, and caspase1 proteins were up-regulated in the decidual tissues of humen and mice in the URSA group. Moreover, the infiltrating cells of decidual tissue in the URSA group were significantly more than those in the control group, the cell arrangement was disordered, and the nucleus was broken. We also found that the increased expression of HMGB1 in the URSA group may come from the active secretion of macrophages. The excessive expression of these proteins could be down-regulated after intervention by the HMGB1 inhibitor aspirin. These data clarify the source and possible mechanism of the high expression of HMGB1 in the decidual tissues of the URSA group, suggesting that HMGB1 and pyroptosis-associated proteins play a vital role in the development of URSA.

The studies about URSA have focused on the influence of peripheral blood immune cells. Recent studies found that the imbalance between Treg and Th17 cells may be related to the pathogenesis of URSA (26), and found that the increase in Th17-related cytokine levels may be one of the related factors of URSA (27). Moreover, the data suggested that the imbalance of the Treg/Th17 ratio may be regulated through HMGB1, and HMGB1 was positively correlated with Th cell-related factors, and negatively correlated with Treg cell-related cytokines (28). HMGB1 structurally contains three functional regions: A box, B box, and acidic C-terminus, and the B box is a functional structural area with two key binding sites for RAGE receptor and Toll-like receptors that play an important role in the inflammatory response (10). The relationship between HMGB1 and diseases has attracted widespread attention, including acute injury-related inflammatory responses, tumors, organ transplants, and autoimmune diseases (29–32). Studies have found that the expression of HMGB1 protein was increased in the villi of RPL patients (14). Our experiment also found that the expression level of HMGB1 was up-regulated in the decidua tissue of URSA patients. These data revealed that HMGB1 may have an association with the pathogenesis of URSA. In addition, we also found that GSDMD, NLRP3, and caspase1 proteins were up-regulated in the decidual tissues of URSA patients, indicating the pathogenesis of URSA may also be related to pyroptosis-associated proteins.

HMGB1 is essential for early pregnancy events before and during blastocyst implantation (12). In the early pregnancy, HMGB1 has been shown to play a key role in driving the proinflammatory response during embryo implantation (16). Exogenous and endogenous HMGB1 initiate an inflammatory response at the time of embryo implantation, high HMGB1 expression around the implantation site, and stromal cell proliferation and differentiation required to induce uterine cell decidualization (13). Several studies have reported that HMGB1 levels are elevated in patients with pregnancy complicated by gestational diabetes mellitus, preeclampsia, and preterm prelabor rupture of membranes (33–35). Our previous results indicated that HMGB1 was highly expressed at the maternal-fetal interface of URSA patients (15, 20). Our study found disorganized cells and cellular infiltration in the mouse decidual tissue of the URSA group, indicating the mouse decidua tissue was in a state of inflammation. However, as a DAMP molecule, HMGB1 plays an important role in proinflammatory (36). Our research results showed that HMGB1 was abundant in the cytoplasm and extracellular in the decidua tissue of the URSA group. Thus, the inflammation at the mouse maternal-fetal interface was induced by HMGB1.

This research indicated that the high level of HMGB1 during pregnancy may induce excessive or continuous inflammation, which will lead to unfavorable pregnancy outcomes at this critical stage of pregnancy. Moreover, during pregnancy, the induction and maintenance of immune tolerance is a prerequisite for a successful pregnancy, involving the participation of a variety of immune cells, such as T cells, NK cells, macrophages, and so on (37). Previous studies have found that decidual immune cells at the URSA maternal-fetal interface play a crucial role in regulating immune tolerance (20). Macrophages can secrete and increase the expression level of HMGB1 in inflammatory response (38). To further investigated the relationship between macrophages and HMGB1, we performed double immunofluorescence staining experiments and found that HMGB1 colocalized with F4/80 or CD68, a surface-specific marker of macrophages, in both human and mouse decidual tissues. Meanwhile, HMGB1 is mainly found in the nucleus and can be actively or passively secreted into the extracellular space to play a pro-inflammatory role (10). Our study indicated that the highly expressed HMGB1 in the URSA group was actively secreted by macrophages.

Previous research found that HMGB1 can be released from immune cells to the extracellular space, increasing sterile inflammation and expanding tissue damage (15), and proposed that it may be regulated by the HMGB1-RAGE/TLR2/TLR4-NF-κB signaling pathway (15). Studies have showed that HMGB1 can be caused acute lung injury by activating inflammasomes in macrophages and was related to TLR2, TLR4, and RAGE/NF-κB signaling pathways (38) HMGB1 can be used as a potential target for bone marrow mesenchymal stem cells to treat multiple organ dysfunction syndromes. The possible mechanism was that HMGB1 may regulate the TLR2, TLR4-mediated NF-κB signal pathway (39). It is well known that HMGB1 can interact with RAGE and Toll-like receptors to participate in cell signal transduction (11). Studies had shown that the TLR4/NF-κB signaling pathway plays an important role in placental inflammation (40). Similarly, the up-regulation of HMGB1 and its receptors RAGE, TLR2, and TLR4 in the URSA group were found in this study, which may suggest that high expression of HMGB1 can induce an increase in the expression of RAGE and Toll-like receptors. Moreover, in the URSA group, there was a strong positive reaction to NF-κB protein in the nucleus and cytoplasm of the decidual tissue, indicating HMGB1 may stimulate the activation of NF-κB through its receptor on URSA.

The activation of the NLRP-3 inflammasome and the increased expression of caspase-1 protein were found in the decidua and chorionic tissues of missed abortion (41). High expression of the NLRP-3 inflammasome was also found at the maternal-fetal interface of patients with recurrent spontaneous abortion, promoting pro-caspase-1 accumulation and division into the activated form caspase-1 when NLRP-3 recognizes stimulatory signals (damage-associated molecular patterns [DAMPs]) *in vitro* or *in vivo*, and then activate IL-1β, IL-18 to participate in subsequent inflammation (42). However, NLRP-3 and caspase-1 are the main participants in the process of pyroptosis, which is a type of programmed cell death closely related to inflammation, mediated by Gasdermin protein, and dependent on caspase activity (43). Moreover, there was research shown that HMGB1 could induce hepatocyte pyroptosis leading to aseptic inflammation and liver damage (19). In this study, HMGB1, NLRP-3, caspase-1, and GSDMD expression levels were up-regulated in the decidua of URSA patients. Meanwhile, these molecules were also highly expressed in the mouse decidua. It was speculated that HMGB1 could induce pyroptosis leading to the onset of URSA.

In recent years, HMGB1 has been widely used as a therapeutic target for diseases (44). HMGB1 was a key pro-inflammatory factor in acute lung injury, involved in the pathogenesis of acute lung injury by inducing M1 polarization through TLR2- and TLR4-mediated NF-κB signaling pathways in macrophages (45). In addition, in hepatic ischemia-reperfusion injury, octreotide and melatonin could reduce the inflammasome-induced pyroptosis by inhibiting the TLR4-NF-κB-NLRP3 pathway (46). Our study found that HMGB1 and their receptors (RAGE, TLR2, TLR4), pyroptosis-associated proteins (NLRP-3, caspase-1, GSDMD), and NF-kB were highly expressed in tissues of unexplained recurrent miscarriage. Besides, studies have shown that taking aspirin for a short period can reduce the level of extracellular HMGB1 (47). Salicylic acid, the hydrolysis product of aspirin *in vivo*, can be used as an effective inhibitor of HMGB1 and applied in the treatment of various diseases such as tumors (48, 49). This hinted that aspirin can be used as a specific inhibitor of HMGB1. Besides, aspirin is a commonly used drug in assisted reproductive technology, especially the treatment of recurrent miscarriage. In our study, mice treated with aspirin reduced their embryonic absorption rate, suggesting the treatment effect of aspirin on URSA. Moreover, our previous study found that low-dose aspirin can reduce the level of HMGB1 in the plasma of URSA patients (15). It was further verified in mouse experiments that the expression level of HMGB1 in the decidua tissue and peripheral blood of the aspirin treatment group was down-regulated. In addition, the expression of RAGE, TLR2, and TLR4 receptors was lower in URSA+ASPL group than that of the URSA group. This study proved that HMGB1 as a therapeutic target of aspirin could down-regulate the level of HMGB1 and its receptors. Meanwhile, this study shown that in the aspirin intervention group, the levels of pyroptosis-associated molecules were down-regulated compared with the URSA group. Therefore, based on previous studies we suggested that HMGB1 might induce pyroptosis through its receptor and NF-κB signaling pathway, cause sterile inflammation, and expand the decidual inflammatory response to mediate the development of URSA, while this inflammatory response can be intervened by the HMGB1 blocker aspirin.

**Figure 7:**
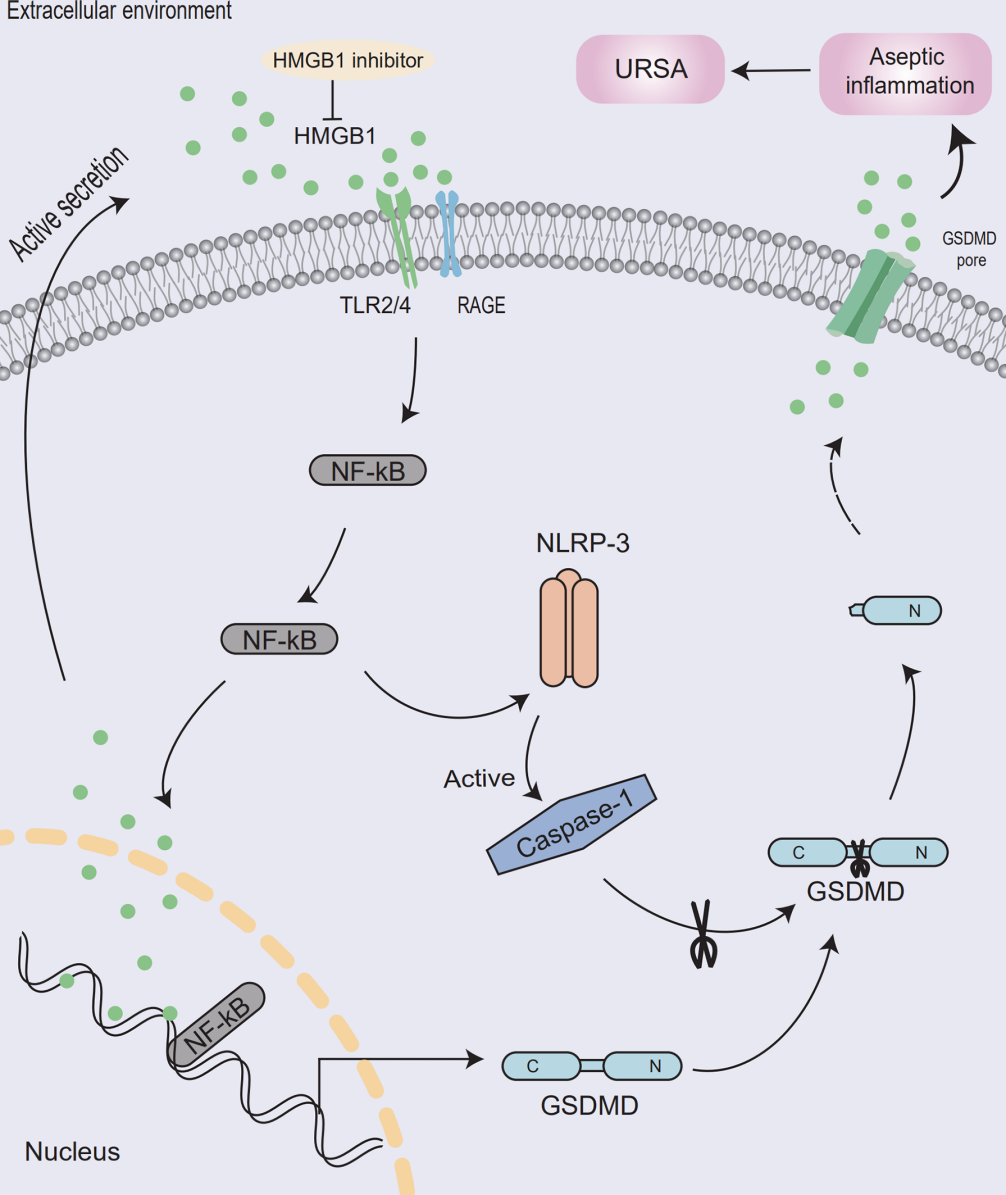
Model of aseptic inflammation caused by HMGB1-mediated pyrolysis in decidua tissue of unexplained recurrent spontaneous abortion. At the maternal-fetal interface, macrophages actively secrete HMGB1 and activate the NF-κB signaling pathway through its receptor. Then NLRP-3 inflammasome will be assembled, activate the caspase-1 protein, and release inflammatory factors such as HMGB1, causing aseptic inflammation at the maternal-fetal interface and leading to the development of URSA. Meanwhile, HMGB1 inhibitor could ameliorates the maternal-fetal interface destruction in unexplained recurrent spontaneous abortion.

In conclusion, the possible mechanism was that HMGB1 entered cells through its receptor and activates NF-κB signaling pathway. Then, pyroptosis would be activated, which induced aseptic inflammation, ultimately leading to the destruction of the maternal-fetal interface and the development of URSA (**Figure 7**). Moreover, aspirin had a therapeutic effect on unexplained recurrent miscarriage. This was the first time to explore the application of HMGB1 as a therapeutic target of aspirin in URSA disease, providing a theoretical basis for the treatment of clinical URSA patients.

## Data Availability Statement

The original contributions presented in the study are included in the article/supplementary material. Further inquiries can be directed to the corresponding authors.

## Ethics Statement

The studies involving human participants were reviewed and approved by the Medical Ethics Committee of the First Affiliated Hospital of Anhui Medical University. The patients/participants provided their written informed consent to participate in this study. The animal study was reviewed and approved by the Experimental Animal Ethics Committee of Anhui Medical University. The animal study was reviewed and approved by the Experimental Animal Ethics Committee of Anhui Medical University (ethics number: LLSC20201138).

## Author Contributions

XX, YC, XW, and HZ designed the study. DZ and HZ performed the experiments and analyzed the data. DZ, HZ, JL, JW, and CM bled the mice and samples acquired. JY and XP helped for language checking. JW, CM, DL, YY, YR, ZZ, and PZ helped for human samples acquired. DZ and HZ wrote the paper. All authors read and approved the final manuscript.

## Funding

The work was supported by grants from the National Natural Sciences Foundation of China (No. 32000642 and 82000399), the Natural Science Foundation of Anhui Province (No.1908085MH244), the Natural Science Foundation of the Anhui Higher Education Institution (No. KJ2019A0285), the Nonprofit Central Research Institute Fund of Chinese Academy of Medical Sciences (No. 2019PT310002), the Research Fund of Anhui Institute of translational medicine (ZHYX2020A001).

## Conflict of Interest

The authors declare that the research was conducted in the absence of any commercial or financial relationships that could be construed as a potential conflict of interest.

## Publisher’s Note

All claims expressed in this article are solely those of the authors and do not necessarily represent those of their affiliated organizations, or those of the publisher, the editors and the reviewers. Any product that may be evaluated in this article, or claim that may be made by its manufacturer, is not guaranteed or endorsed by the publisher.
